# Complete Genome Sequence of the Endophytic Fungus *Diaporthe* (*Phomopsis*) *ampelina*

**DOI:** 10.1128/genomeA.00477-16

**Published:** 2016-06-02

**Authors:** J. Savitha, S. D. Bhargavi, V. K. Praveen

**Affiliations:** Department of Microbiology and Biotechnology, JB Campus, Bangalore University, Bangalore, India

## Abstract

*Diaporthe ampelina* was isolated as an endophytic fungus from the root of *Commiphora wightii,* a medicinal plant collected from Dhanvantri Vana, Bangalore University, Bangalore, India*.* The whole genome is 59 Mb, contains a total of 905 scaffolds, and has a G+C content of 51.74%. The genome sequence of *D. ampelina* shows a complete absence of lovastatin (an anticholesterol drug) gene cluster.

## GENOME ANNOUNCEMENT

Lovastatin (C_24_H_36_O_5_) is an anticholesterolemic drug approved by the U.S. Food and Drug Administration (FDA) in 1987. It acts as a competitive inhibitor of the enzyme 3-hydroxyl-3-methylglutaryl coenzyme A (HMG-CoA) reductase in cholesterol biosynthetic pathway, and therefore, it is prescribed for the treatment of hyperlipidemia. Several filamentous fungi belonging to the genera *Aspergillus*, *Penicillium*, *Monascus*, and *Pleurotus* are reported to be lovastatin producers. However, *Aspergillus terreus* is being used for the commercial production of lovastatin (1).

Endophytic fungi are the proven sources of secondary metabolites with pharmaceutical importance. Therefore, they are exploited to grow as axenic cultures on synthetic medium under controlled conditions (devoid of interaction with host plant) for the production of commercially valuable secondary metabolites with anticancer, antioxidant, anti-inflammatory, antiparasitic, antiviral, and antimicrobial properties. However, there are very few reports on the production of lovastatin by endophytic fungi (2, 3). The results of our previous biochemical (4), bioinformatics (5), and molecular-level studies (6) with soil and endophytic fungi have authentically concluded that most, if not all, of the endophytic fungi are the potential candidates for lovastatin production. *Diaporthe*, previously known as *Phomopsis* (7), represents frequent isolates of tropical and temperate medicinal plants (4, 8) and is taken as a model endophytic fungus for our present study.

*Diaporthe ampelina* was cultured on potato dextrose broth (PDB) at 28°C (pH 6.0) and 120 rpm for 7 days. DNA was extracted by use of a cetyltrimethylammonium bromide (cTAB) method (9). Quality of DNA was checked on 1% agarose gel (5 µl loaded) for the single intact band. The gel was run at 110 V for 30 min. One microliter of sample was loaded in a NanoDrop 2000 to determine the *A*_260_/*A*_280_ ratio, and 1 µl of sample was used to determine concentration using a Qubit 3.0 fluorometer.

Whole-genome sequencing of *D. ampelina* was carried out using a HiSeq 2500 with 2 × 125-bp chemistry. A total of 17,737,682 reads (4.4 Gb) were generated. The raw data were quality filtered using Trimmomatic version 0.35. A total of 4.23 Gb of high-quality data with 17,006,041 reads were obtained and used for downstream analysis. High-quality reads were assembled using ABySS (version 1.5.2) and SSPACE (version 3.0); as a result, 59 Mb in 905 scaffolds were assembled, with an *N*_50_ of 134,716 bp. A total of 24,672 genes were predicted using Augustus (version 3.2.1). Out of 24,672 genes, 20,727 genes were annotated, while 3,945 genes were not annotated against the NR database during functional annotation performed using BLASTx. None of the genes of lovastatin biosynthesis clusters (AF141924.1 and AF141925.1) were aligned on the scaffolds. There was a complete absence of a lovastatin biosynthetic gene cluster in the whole genome of *D. ampelina.*

### Nucleotide sequence accession number.

This complete genome sequence has been deposited at DDBJ/GenBank/EMBL under accession no. LWAD00000000.
